# Prevalence and determinants of diabetic polyneuropathy and diabetic foot complications in a specialized clinic

**DOI:** 10.1186/1758-5996-7-S1-A42

**Published:** 2015-11-11

**Authors:** Marcos Oliveira Pires de Almeida, Leyna Leite Santos, Denise Dantas Lima, Taciana Borges Cavalcanti, Amanda Aleixo, Vanessa Silva de Almeida, Diego de Sousa Dantas, Bárbara Bernardo Silva, Evandro Cabral de Brito, Paulo André Freire Magalhães

**Affiliations:** 1 Universidade Federal de Pernambuco, Recife, Brazil

## Background

Diabetic peripheral neuropathy is one of the common chronic complications of diabetes and a cause of limb amputations. Foot complications are considered to be a serious consequence of diabetes mellitus. At the time of diagnosis, more than 10% of people with type 2 diabetes mellitus have one or two risk factors for foot ulceration and a lifetime risk of 15%. Identifying the extent of this problem and its risk factors will enable health providers to set up better prevention programs.

## Objective

The objectives of this study were to determine the prevalence of peripheral neuropathy and diabetic foot complications, describe the clinical features and identify risk factors diabetic patients.

## Materials and methods

A cross-sectional study was conducted at Pernambuco Unit of Specialized Care in the city of Limoeiro. Participants included 216 patients with type 2 diabetes treated at unit from May 2014 to April 2015. All completed an interviewer-administered questionnaire and underwent medical assessment including foot examination and assessment of presence of peripheral sensory neuropathy (PSN). The patients were assigned to a foot risk category which was developed by the International Working Group on the Diabetic Foot (IWGDF).

## Results

A total of 97 males (45%) and 119 females (55%) were included. The mean (standard deviation) values were 54.5 (10,4) yrs. for age and 7.8 (7,3) yrs. for diabetes duration. The prevalence of PSN was 71.3%. The polyneuropathy was symptomatic in 110/154 (71.4%) patients. Diabetes duration, age, hypertension, use of insulin and low family income were significantly associated with PSN (p < 0.05). Foot deformity was noticed in 86 patients (40%), 40 (18.5%) patients had a history of ulceration and five patients had amputation of limbs. According to the modified IWGDF classification, 27.8% of all patients were considered as having low-risk (group 0), and 57.9% of the study population were at high risk for pedal ulceration (group 2 and 3).

## Conclusions

The prevalence of PSN and high risk for pedal ulceration were very high in this study, demonstrating that diabetes is not adequately controlled and even culminates in chronic complications. This emphasizes the importance of implementing simple and affordable screening tools and methods to identify high-risk patients and providing foot care education for them as well as treat and control the disease more effectively.

